# Swall-E: A robotic in-vitro simulation of human swallowing

**DOI:** 10.1371/journal.pone.0208193

**Published:** 2018-12-19

**Authors:** Yo Fujiso, Nicolas Perrin, Julian van der Giessen, Nihal Engin Vrana, Fabrice Neveu, Virginie Woisard

**Affiliations:** 1 PROTiP Medical SAS, Strasbourg, France; 2 Voice and Deglutition Unit, Department of Otorhinolaryngology and Head and Neck Surgery, Larrey Hospital, Toulouse, France; Istituto Italiano di Tecnologia Center for Micro BioRobotics, ITALY

## Abstract

Swallowing is a complex physiological function that can be studied through medical imagery techniques such as videofluoroscopy (VFS), dynamic magnetic resonance imagery (MRI) and fiberoptic endoscopic evaluation of swallowing (FEES). VFS is the gold standard although it exposes the subjects to radiations. In-vitro modeling of human swallowing has been conducted with limited results so far. Some experiments were reported on robotic reproduction of oral and esophageal phases of swallowing, but high fidelity reproduction of pharyngeal phase of swallowing has not been reported yet. To that end, we designed and developed a robotic simulator of the pharyngeal phase of human swallowing named Swall-E. 17 actuators integrated in the robot enable the mimicking of important physiological mechanisms occurring during the pharyngeal swallowing, such as the vocal fold closure, laryngeal elevation or epiglottis tilt. Moreover, the associated computer interface allows a control of the actuation of these mechanisms at a spatio-temporal accuracy of 0.025 mm and 20 ms. In this study preliminary experiments of normal pharyngeal swallowing simulated on Swall-E are presented. These experiments show that a 10 ml thick bolus can be swallowed by the robot in less than 1 s without any aspiration of bolus material into the synthetic anatomical laryngo-tracheal conduit.

## Introduction

### Swallowing

**Fig 1:**
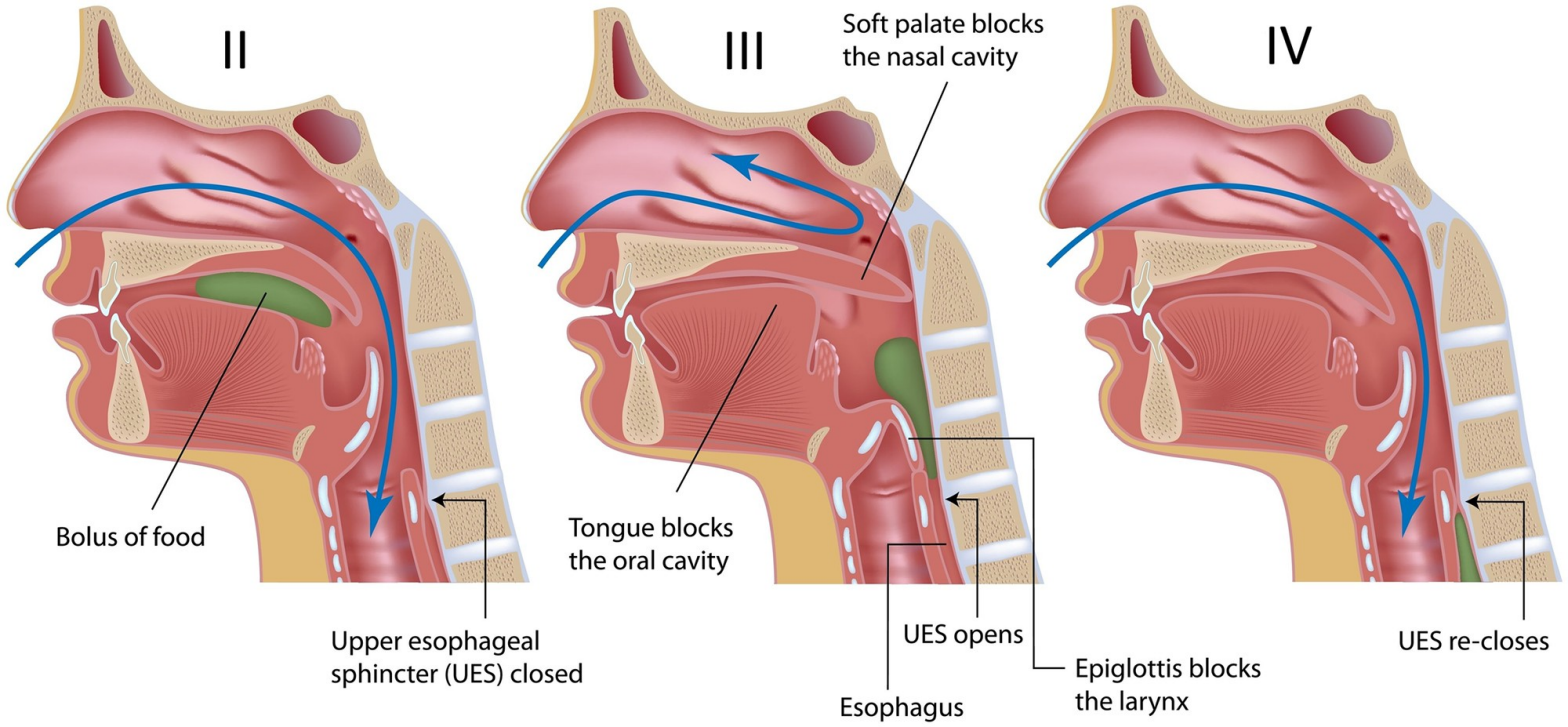
Schematic representation of phases II, III and IV of human swallowing.

Swallowing is a fundamental physiological function whereby food and liquids are transported in a synchronized and sequential manner from the oral cavity to the esophagus, passing through the pharynx, also known as the aerodigestive crossroads. Swallowing can be divided into (I) the preparatory phase, (II) the oral phase, (III) the pharyngeal phase and (IV) the esophageal phase [1]. Phases II, III and IV are schematically represented on Fig 1 for better comprehension. A bolus, i.e. foods and/or liquids mixed with saliva, is masticated and shaped in the oral cavity during the preparatory phase. The bolus is then propelled by the tongue from the oral cavity to the pharynx during the oral phase. During the pharyngeal phase, the bolus transits the pharyngeal cavity and is transported towards the esophagus. The pharynx is divided into three regions, from top to bottom: nasopharynx; oropharynx; hypopharynx. Bolus normally flows only from the oropharynx to the hypopharynx, while the lower airways, i.e. the larynx, the trachea and the lungs, are protected during the pharyngeal phase by a number of protective reflex mechanisms [1], mainly the laryngeal elevation and the sequential closure of laryngeal anatomical structures: vocal folds, ventricular folds, aryepiglottic folds and epiglottic fold [2]. Once the swallowed bolus safely reaches the esophagus, by passing through the open upper esophageal sphincter (UES), the esophageal phase of swallowing commences, whereby the bolus is conveyed downwards (down to the stomach) thanks to the peristaltic motion of the esophagus. While normal (healthy) swallowing ensures a safe transport of foods and liquids by preventing them from entering the airways during the pharyngeal phase, in case of disrupted (pathological) swallowing, which is called dysphagia, part or all the bolus may accidentally enter the trachea e.g. due to disrupted timings of events (desynchronizations or delays of mechanisms) and/or impaired/insufficient protective mechanisms.

### Robotic simulation of swallowing

Robotic systems for partially simulating different phases of swallowing were previously developed. Woda et al [3] developed a mastication simulator to observe food bolus formation during mastication. Doyennette et al [4, 5] created a mechanistic model to study the rheological effects of masticated foods and viscous Newtonian liquids on aroma and flavor release during the oral phase. Mackley et al [6] and later Hayoun et al [7] worked on an arm and roller based mechanical system to model the oral swallowing of Newtonian fluids, named the ‘Cambridge Throat’. Their work provided preliminary results about the relationship between the physical properties of the bolus and the bolus transit time. With a particular focus on the control of the actual peristaltic motions by esophagus, Zhu et al [8] developed a robot to mimic the esophageal peristaltic movement which can convey the introduced food material via peristaltic wave signals generated by a central pattern generator. Dirven et al [9] recently used a robot for rheological investigations of food bolus and demonstrated that predictions based solely on bolus viscosity can be misleading, showing the potential benefits of robotic testing systems. The swallowing robot from Noh et al [10] includes an artificial head composed of mandible, tongue, pharynx, larynx, epiglottis, trachea, which is able to mimic swallowing motions based on VFS data. The main drawback of this system is its absence of actuation of the epiglottis and the pharynx, making it more suitable to study the oral phase of swallowing than the pharyngeal phase.

Regarding the pharyngeal phase, specifically, Stading and Qazi are currently developing a mechanical in-vitro apparatus named the ‘Gothenburg Throat’ [11] which aims at investigating the rheology of bolus during the pharyngeal phase. This apparatus consists of a duct assembly of simplified rigid geometries representing the tongue, pharynx, larynx, trachea, epiglottis and esophagus. Tested boli are injected by a motorized syringe inside the duct. Furthermore, this apparatus is equipped with ultrasonic velocimetry and pressure sensors positioned at relevant locations of the duct to allow precise rheological characterization of the tested bolus flow in a controlled way. The main limitation of such a device is the rigidity of its anatomical structures which cannot deform.

### Aim of this study

Our aim was to develop an in-vitro mechatronic system that can realistically simulate the pharyngeal phase of human swallowing, in terms of both physiology and anatomy. This system should therefore be able to swallow an injected bolus from the pharyngeal inlet down to the upper esophagus inlet (i.e. UES) in timings similar to those of a real pharyngeal swallowing, i.e. in a time interval of less than 1 s [1]. Moreover, due to the importance of the interaction between swallowing and respiration with regards to the swallowing efficiency [2], which is still far from being clearly understood [12], and the lack of such an important physiological function in other existing swallowing robots, our system is designed to simulate a respiratory airflow. To that end, we created a robotic apparatus, named Swall-E, based on a realistic model of a human pharyngo-laryngeal tract in which the reproduced anatomical structures and mechanisms involved in the pharyngeal swallowing phase can be precisely actuated and controlled.

## Materials and methods

### Swall-E robot

#### Design and specifications

**Fig 2:**
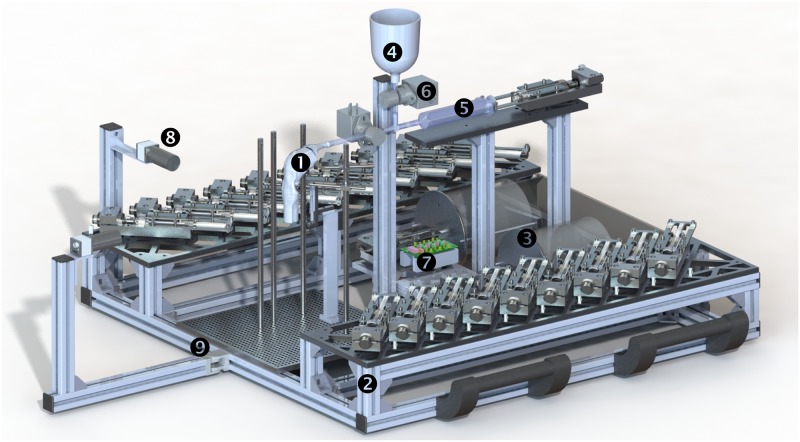
**Overview of Swall-E swallowing robot**—actuation wires, electrical cables and power supply non depicted: (1) synthetic anatomical conduit; (2) actuator; (3) pressure generator; (4) bolus storage tank; (5) motorized syringe; (6) solenoid valve; (7) electronic card board and microcontroller; (8) lateral high-speed camera; (9) aluminum frame.

Swall-E swallowing robot, illustrated in Fig 2, was designed with the main objective of mimicking the anatomy and physiology of the pharyngeal phase of adult human swallowing, as faithfully as possible, in terms of dynamics, timings and dimensions. Thus, the main starting specifications were as follows: real scale model of adult human anatomical tract in optimally chosen soft material; reproduction of anatomical structures directly involved in the bolus transport process occurring in the pharyngeal phase; reproduction of critical physiological mechanisms in a precise and adjustable manner, spatially and timely. Such reproduced mechanisms are: bolus injection by base of tongue movement; vocal fold opening/closure; laryngeal elevation; pharyngeal contraction; epiglottic closure of larynx; UES opening/closure; respiration. The overall architecture of Swall-E is centered around a synthetic anatomical conduit, which is set in motion by an actuation system. The overall assembly is mounted on an aluminum frame of dimensions 850 × 850 mm. A power supply provides electricity to all the electromechanical components.

#### Anatomical conduit

**Fig 3:**
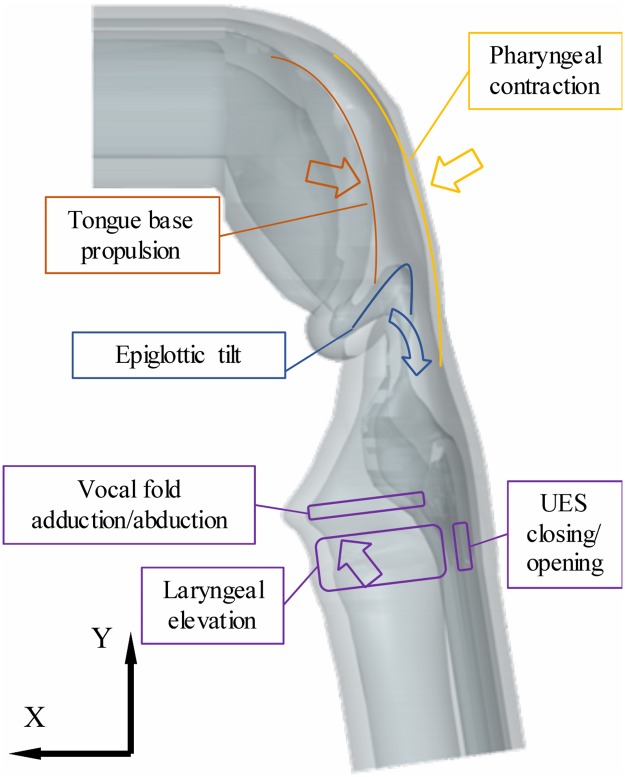
Side view of Swall-E synthetic anatomical conduit.

The synthetic anatomical conduit (Fig 3) is derived from a computer aided design (CAD) geometry generated from a computerized tomography (CT) scan of a human aerodigestive tract (healthy male subject between 20 and 25 years of age). The following anatomical structures are included in this conduit: base of tongue; oropharynx; hypopharynx; epiglottis; larynx and upper part of trachea (30 mm); upper part of esophagus (30 mm) including the UES. In this first version, we decided not to incorporate a velopharyngeal sphincter and not to reproduce the velopharyngeal closure mechanism, as their contribution to laryngo-tracheal aspiration mechanisms occurring in the laryngeal and hypopharyngeal regions is limited. Thus, the anatomical conduit is closed at the velopharyngeal region.

#### Materials

For the anatomical conduit, efforts were made to select optimal soft materials with mechanical properties as similar as possible to those of the real anatomical structures and tissues. In order to simplify the manufacturing process of the conduit, we decided to use only a single soft material. The different hardnesses of the various anatomical structures were taken into account by locally varying the thickness of the material according to the local anatomical region (tongue, pharynx, larynx, esophagus). Silicone, due to its mechanical behavior suitable for this type of biomechanical application [13, 14] and translucency allowing some internal visualization, was selected. The hardness of the selected silicone was determined by having several silicone samples of different hardness levels independently evaluated by seven experienced ENT surgeons. Of all the evaluated samples, the 13-Shore A hardness sample was unanimously deemed the most suitable. The manufacturing process of the silicone anatomical conduit involved silicone injection molding in a 3D-printed mold created from the aero-digestive CAD model.

#### Actuation system

**Fig 4:**
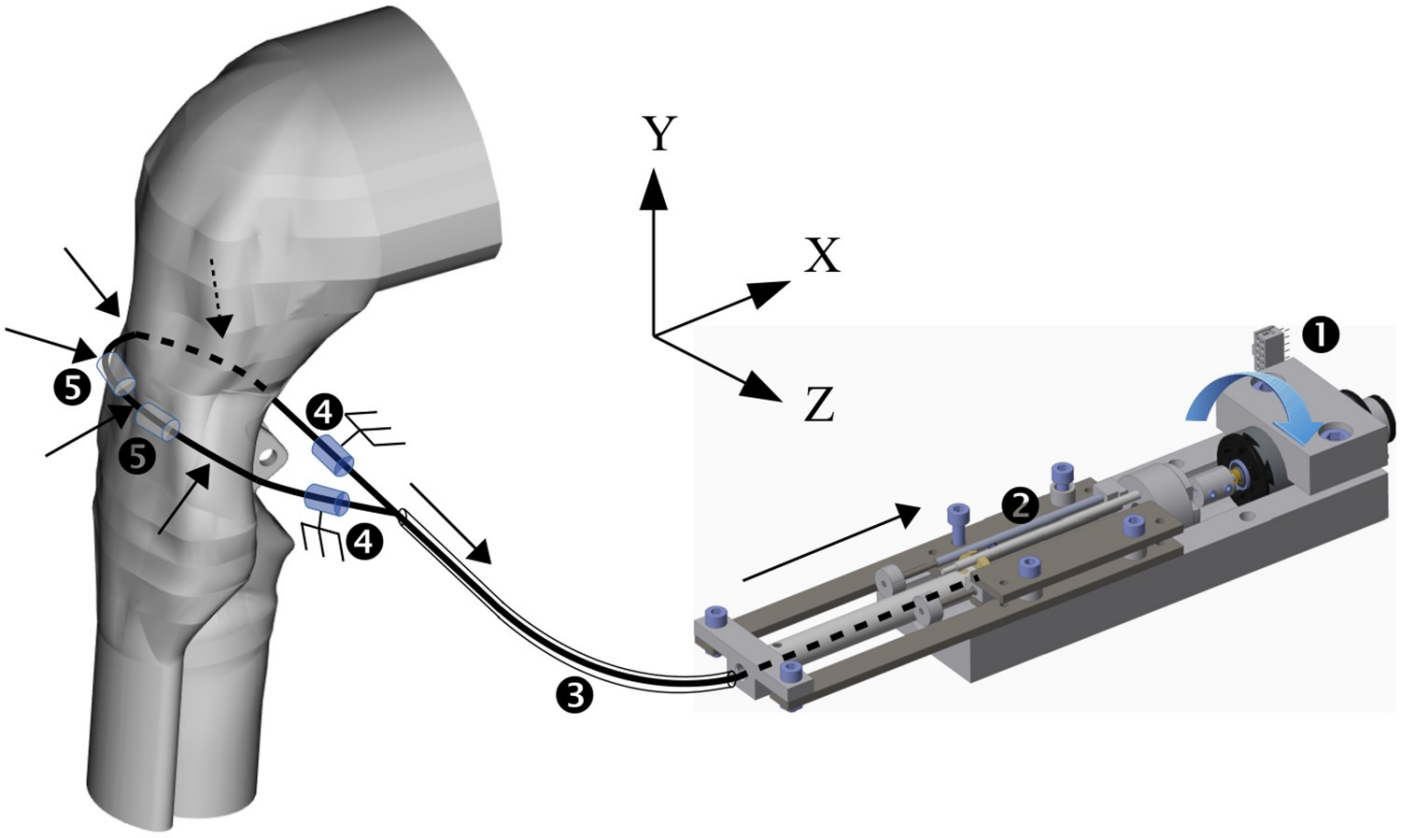
Deformation of anatomical conduit by metal wire-based actuation. Arrows represent mechanical forces and their drawn directions correspond here to a swallowing sequence onset. (1) electrical motor + optical coder; (2) screw-nut system; (3) plastic sheath; (4) wire guides; (5) transparent pads.

The sequenced and synchronized motions of the reproduced mechanisms are achieved by an actuation system (of spatial accuracy 0.025 mm and temporal accuracy 20 ms) based on metal wires directly deforming the silicone conduit (Fig 4). This type of actuation system was chosen over other systems (e.g. hydraulic based or mechanical roller based systems) owing to its robustness, precision and relative technical ease of manufacturing. 17 actuators pull and push the metal wires. Each actuator is made of an electrical motor (ref. Maxon DC-MAX26S EB KL 12V) with an embedded 32-increment optical coder (ref. Maxon ENX16 EASY 32IMP) attached to a screw-nut system of length 50 mm and screw thread of 0.8 mm (hence the spatial accuracy of 0.025 mm = 0.8/32). Each nut is attached by a pressure spindle to a 1 mm-diameter galvanized steel wire (ref. 1 mm diameter STANDERS lifting cable). This type of wire was selected due to its suitable flexibility for this application. Each wire is covered by a plastic sheath of inner diameter 1.1 mm to ensure a reversible movement of the wire regardless of a tensile or compressive stress exerted to it. The sheath prevents excessive bending of the wire. Sheath proximal ends (close to the anatomical conduit) are supported by spatially adjustable metal rods screwed on the aluminum frame. Wires are guided by 3D-printed transparent pads glued on the silicone conduit. The wires and pads are positioned so that to mimic the muscular insertions of head and neck muscles involved in the pharyngeal swallowing process. Symmetrical pair muscular insertions are mimicked by curving a wire of which both ends are symmetrically attached to one actuator.

#### Tongue, pharynx and larynx movements

**Fig 5:**
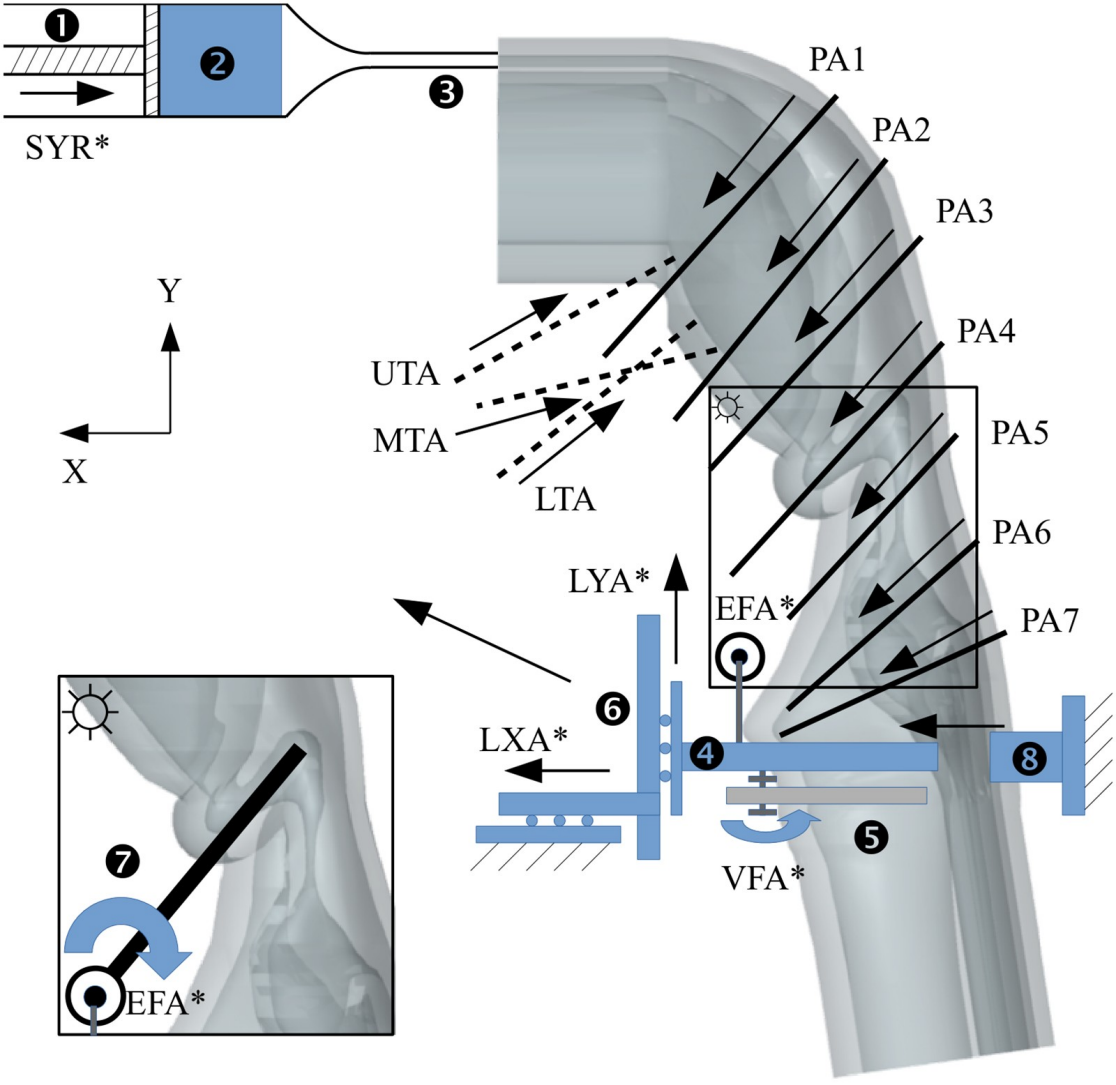
List of actuator (linear and rotational) connections and corresponding labels. (1) syringe piston; (2) bolus material; (3) injection nozzle; (4) ‘cricoid’ piece; (5) vocal fold pinching clamp; (6) horizontal and vertical slides; (7) epiglottic metal stem; (8) upper esophagus connection to aluminum frame. Solid black lines represent the pharynx actuation wires while dotted lines represent the tongue actuation wires. * stands for non curved actuation wires. The whole actuation system of the epiglottis (e.g. shaft and transmission belts) is not represented.

In physiological swallowing, the bolus is propelled by the base of tongue against the posterior pharyngeal wall at the beginning of the pharyngeal phase [1]. Then, the pharyngeal posterior muscles which are mainly the three pharyngeal constrictor muscles (upper, middle and lower) exert a continuous and smooth constricting movement to the bolus in order to progressively transport it towards the esophagus [1, 15]. Simultaneously, the larynx elevates in the anterior and superior directions to protect the airways. On Swall-E, only the base of tongue is reproduced (i.e. not the complete tongue) as it is the most important part of tongue involved in pharyngeal swallowing and it enables increased amplitude of backward propelling movement. The propulsion movement of this synthetic base of tongue is achieved by three wires passing through pads positioned on the curved anterior side of the base of tongue. The pharyngeal contraction movement is induced by seven wires passing through pads positioned on the curved posterior side of the pharynx. Laryngeal elevation movement is achieved by two slides mounted perpendicularly in the horizontal (X) and vertical (Y) directions (Fig 5), which are rigidly attached to a rigid 3D-printed piece acting both as a cricoid cartilage and a hyoid bone. The X and Y directions correspond respectively to the physiological anterior-posterior and inferior-superior directions. The two slides are also attached to two wires and corresponding actuators. The so-called cricoid piece itself is attached to the laryngo-tracheal duct to pull it in the X and Y directions during the laryngeal elevation.

#### Anatomical folds and sphincters

**Fig 6:**
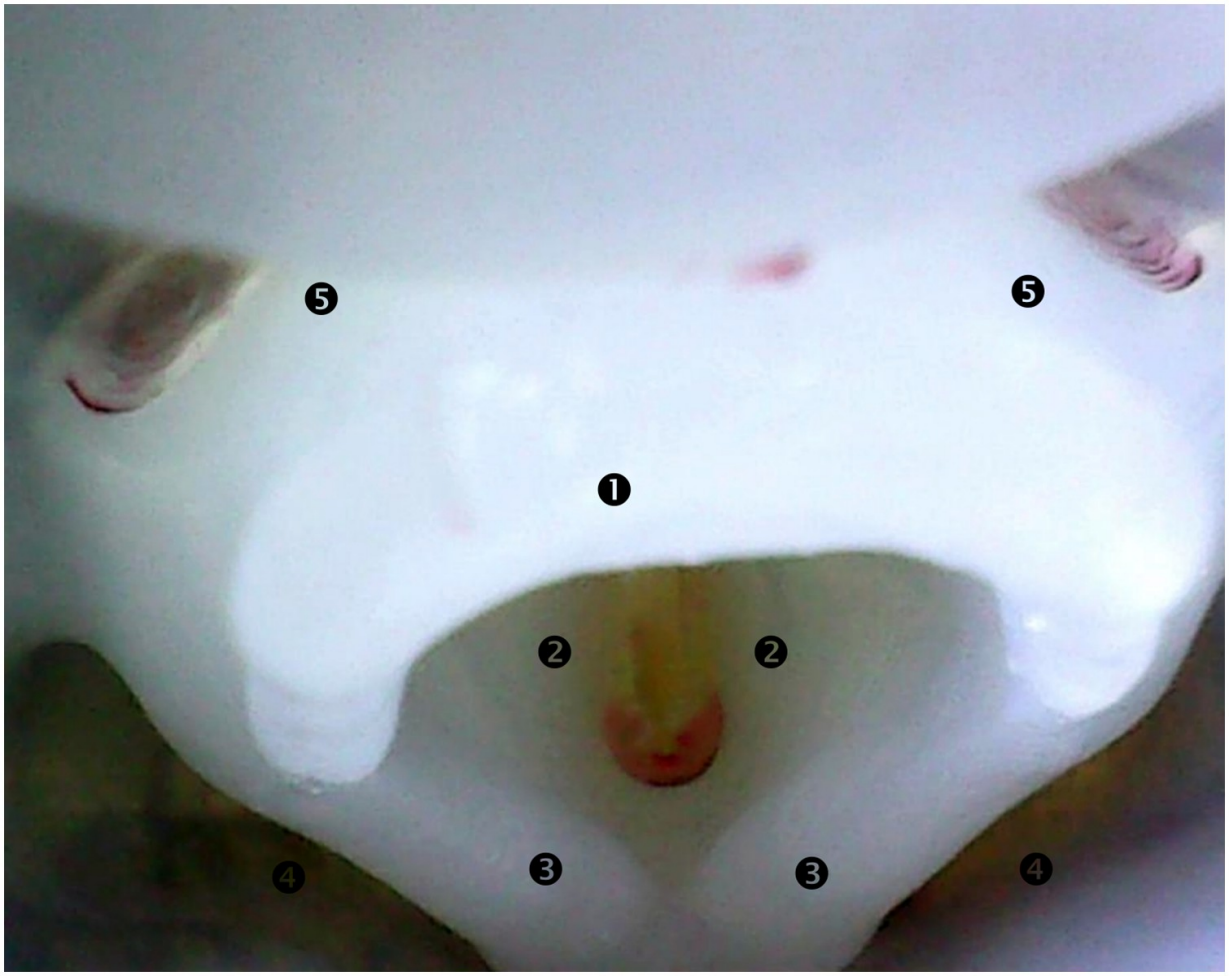
Endoscopic view of Swall-E pharyngeal cavity. (1) epiglottis; (2) vocal folds (glottis); (3) aryepiglottic folds; (4) piriform sinus; (5) valleculae.

The actuated anatomical folds and sphincters are the vocal folds, the epiglottic fold and the UES. Vocal folds are already shaped in the silicone anatomical conduit (Fig 6). The vocal fold abduction/adduction (or opening/closure) mechanism is handled by a clamp pinching the laryngo-tracheal duct (Fig 5) at the vocal fold plane (or glottic plane). The jaws of the clamp open and close the two synthetic vocal folds thanks to a horizontal pulling/pushing wire. The epiglottis is modeled by a silicone flap also already shaped in the silicone conduit (Fig 6), partially overmolded on a guiding metal stem (the tip of the synthetic epiglottis remains entirely flexible and free to move, though). While in physiological reality the epiglottic tilt movement is passive (i.e. non activated by muscles), induced by the coordination of laryngeal elevation and approximation of hyoid bone and thyroid cartilage [16], in Swall-E it is actively actuated (Fig 5) in order to have more control over it in case of pathological swallowing modeling. The epiglottic actuation is mechanically performed by connecting the metal stem to a shaft, the latter being attached to an electrical motor through two universal joints and two symmetrical transmission belts. Such a mechanism allows three degrees of freedom: one rotation in the (XY) plane, and two translations in the X and Y directions. As for the UES, its opening/closing pattern, having also critical physiological impact on the swallowing function efficiency [17, 18], is created on Swall-E by having the synthetic cricoid cartilage (on its posterior surface) glued to it (Fig 5). By pulling anteriorly the synthetic cricoid cartilage during laryngeal elevation movement, the UES opens accordingly. At rest, the UES remains closed like in physiological reality [18].

### Control and instrumentation

#### Control system

Swall-E actuation system is piloted by an electromechanical control system. The rotational position of each motor, thus the linear position (i.e. displacement) of each nut, is independently piloted by a 2nd order servo position controller. A microcontroller (ref. Microchip PIC24EP) is in charge of piloting all the actuators during a 10 s sequence, with a temporal spacing of 20 ms (i.e. a set of 500 consecutive position values), hence the previously mentioned temporal accuracy of 20 ms. This temporal spacing is due to the limitations of the microcontroller. Programed chronological displacement sequences of actuators are referred thereafter by ‘chronograms’. A LabVIEW (National Instruments, Inc.) graphical user interface (GUI) was developed to allow precise control and settings of all the actuators and other electromechanical components of the robot. The GUI imports text files containing preprogramed 500 point-chronograms and sends them to the microcontroller, to be played by the actuators. It is therefore possible to reproduce the complex coordinated pattern of mechanisms occurring in a pharyngeal swallowing process by preparing chronogram text files beforehand. This preparation can be either done manually on the GUI (through displacement graphs manually editable) or more automatically using a programming tool that can produce output text files, e.g. MATLAB (Mathworks Inc.).

#### Control of pressure

Another important feature is the dynamic control of pressure (i.e. pressure generation) inside the laryngo-tracheal duct and inside the esophageal duct. This feature is carried out by two identical pressure generators, each one consisting in a piston mounted in a plexiglas cylinder of length 15 cm and diameter 13 cm associated with a pressure transducer (ref. NovaSensor NPC-100) which monitors the pressure at the outlet of the cylinder. The outlet pressure dynamically generated by the piston is piloted by a servo control which, according to the set pressure chronogram, dynamically adjusts the piston position. A solenoid valve (ref. Bürkert 6213 EV) is mounted downstream of the piston outlet to either completely shut or allow the airflow generated from the piston. A vent tube is connected to the conduit in the velopharyngeal region at one end and at a solenoid valve (ref. SMC VDW 10AA) at the other end leading to ambient air, to act as a respiratory ‘nose’. The capability of pressure generation in the laryngo-tracheal duct enables a controlled respiratory airflow, useful to study for instance the coordination between swallowing and respiration, critical on swallowing function outcome [2].

#### Bolus injection

The bolus injection mechanism into the anatomical conduit is carried out by a 60 ml motorized syringe. The bolus fluid material is stored inside a tank installed upstream of the motorized syringe through a T-shaped tube. Two pinch solenoid valves (ref. Fluid Concept S126) are positioned in the injection circuit to precisely control the bolus injection sequence, which occurs as follows: (1) the syringe aspirates a set volume of bolus from the bolus tank; (2) the syringe propels the set volume of bolus into the conduit at a predefined injection timing through a nozzle air-tightly connected to the conduit (Fig 5); (3) the syringe returns to its initial position. This system is able to achieve an injection of 10 ml of liquid or thick bolus in 100 ms, i.e. an injection flowrate of 0.1 l/s.

#### Instrumentation

In terms of instrumentation, Swall-E is equipped with various cameras for visual monitoring and video recording of the simulated swallowing events occurring inside the translucent silicone anatomical conduit. Two high-speed cameras (ref. iDS UI-3160CP-C-HQ Rev.2), allowing synchronized video recordings at 100-300 frames per second (FPS), are positioned 30 cm from the anatomical conduit in lateral and rear sides of it. Two generic USB endoscopic cameras of diameter 5.5 mm can be inserted in the laryngo-tracheal duct and the esophageal duct to enable endoscopic visualization below the vocal folds and the UES. A third endoscopic 5.5 mm camera is mounted on the posterior pharyngeal wall to allow endoscopic visualization of the pharyngeal cavity.

## Results and discussion

### Simulated swallowing experiments

**Fig 7:**
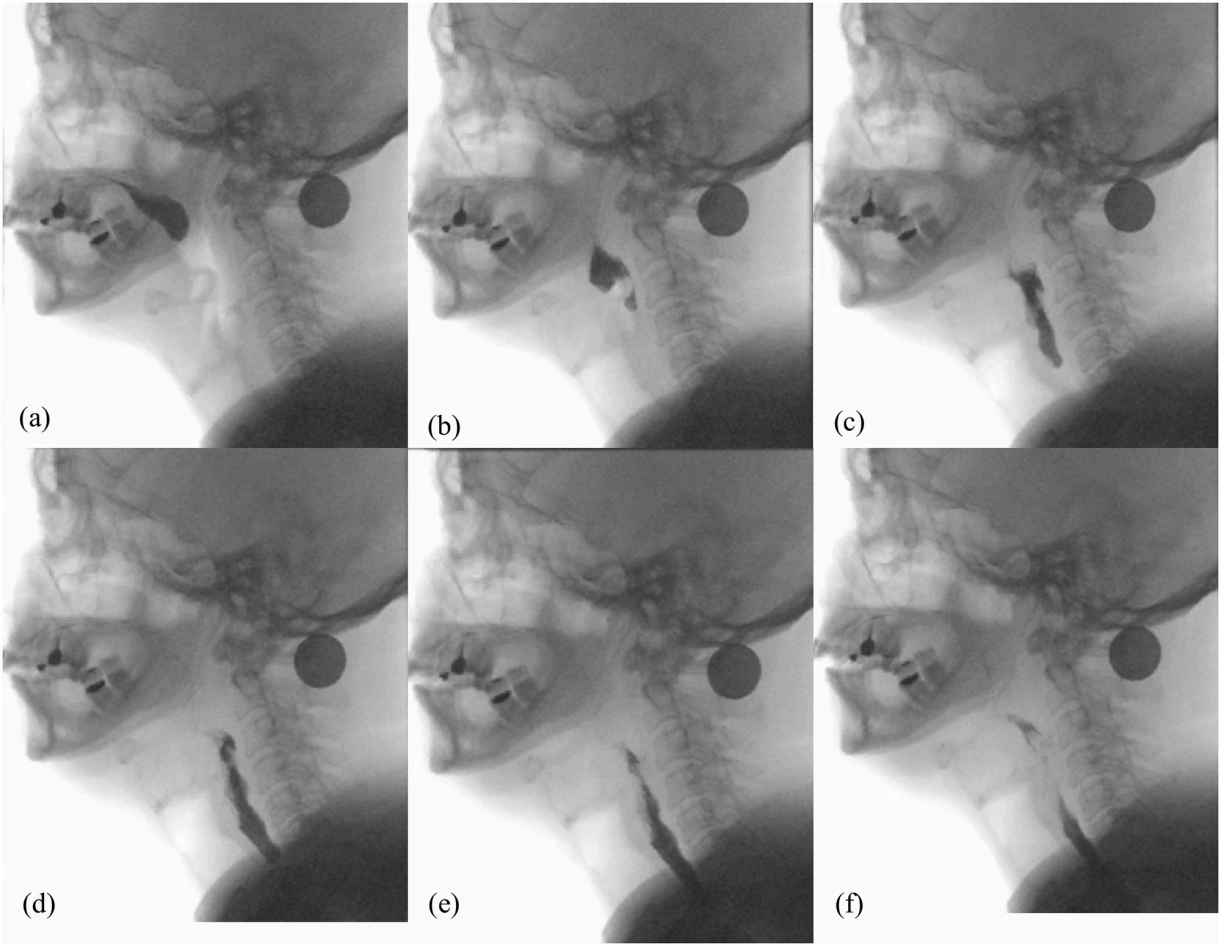
Successive VFS images of a normal pharyngeal phase of swallowing recorded from an around 50 years old subject (3 ml thick bolus of custard type texture). (a) [0 ms] Bolus pushed by tongue, entering the pharyngeal cavity. (b) [133 ms] Bolus at oropharyngeal level above epiglottis; larynx elevated. (c) [200 ms] Bolus squeezed by pharyngeal contraction; epiglottis tilted. (d) [267 ms] Bolus entering the open UES and the esophagus. (e) [400 ms] Continuation of pharyngeal contraction. (f) [533 ms] Bolus conveyed downwards by esophageal peristalsis; larynx descended. Note: the black disk is a 0.5 € coin serving as a fiducial marker.

Simulated swallowing experiments were conducted to demonstrate the fundamental capabilities of Swall-E robot. We simulated a normal (i.e. non pathological) pharyngeal swallowing cycle based on in-vivo timings reported in normal swallowing studies. Our aim was to achieve a transit of injected bolus through the pharynx in less than 1 s, without any aspiration of bolus material into the laryngo-tracheal duct. For reference, Fig 7 shows some successive VFS images of a normal pharyngeal swallowing recorded from an around 50 years old subject, swallowing a 3 ml thick bolus of custard type texture. These data were retrospectively obtained from previously-collected data, and were anonymized prior to the current study. Proper formal written consent from the subject to utilize these data for education or research purposes was obtained prior to the data collection.

### Bolus material

A bolus material of thick consistency was prepared prior to the experiments, by pouring and mixing one spoonful of thickening powder (ref. Nutricia Power, Nutilis) into 200 ml of still water, and adding 15 droplets of red food coloring to improve visibility of the bolus. This type of thickening alimentary powder is commonly utilized by dysphagic patients to help them swallow liquids more easily by increasing the viscosity of the swallowed liquids. We used the IDDSI (International Dysphagia Diet Standardisation Initiative [19]) protocol to characterize the texture of the bolus in a standardized way. According to this protocol, the tested bolus was a class 3 texture i.e. a moderately thick texture (0 corresponding to thin textures and 4 to purees).

### Experimental protocol

**Fig 8:**
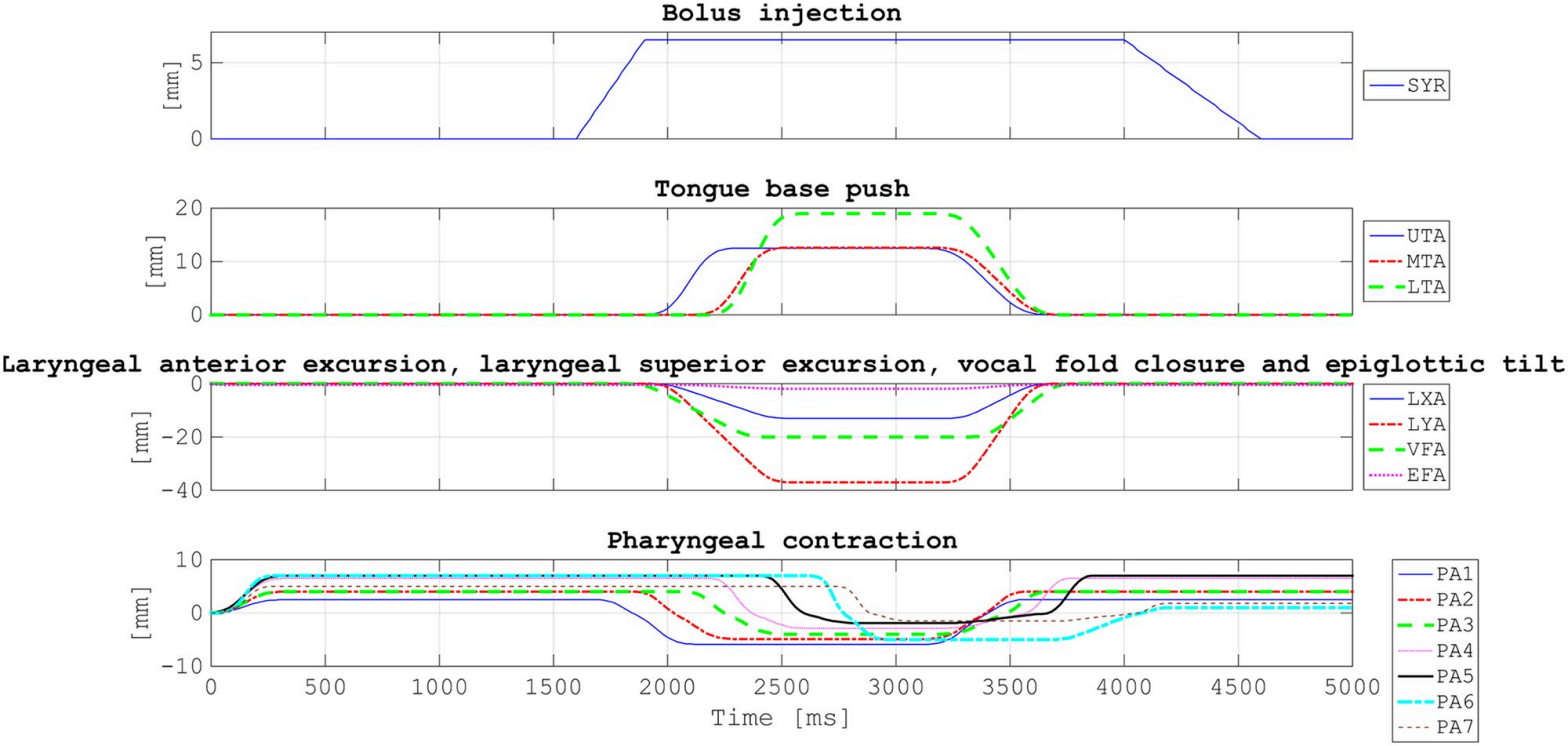
Actuation chronograms mimicking a normal pharyngeal swallow generated in MATLAB. SYR = syringe; UTA = upper tongue actuator; MTA = middle tongue actuator; LTA = lower tongue actuator; LXA = laryngeal anterior actuator; LYA = laryngeal superior actuator; VFA = vocal fold actuator; EFA = epiglottic fold actuator; PA*i* (*i* ∈ [1; 7]) = pharyngeal actuator *i* (from top to bottom). Notes: While the epiglottic actuator delivers only a rotational output instead of a linear output, its actuation chronogram is represented here by a linear displacement in mm like for other actuators (by conversion of number of rotational increments into corresponding linear distance using the screw thread value of the screw-nut systems of the other actuators). The UES opening is coupled to the LYA actuation.

A 10 ml volume of the prepared bolus material was stored by the motorized syringe to be injected in the anatomical conduit at a flow rate of 0.1 l/s. In these experiments no respiration was simulated in order to focus solely on the pharyngeal swallowing process. One single cycle of pharyngeal swallowing was simulated and simultaneously filmed by the rear and the lateral high-speed cameras at 105 FPS. After the actual swallowing cycle, all the actuators and the conduit were automatically reset to their original positions. The actuation chronograms utilized for these experiments are depicted on Fig 8. These chronograms were generated in MATLAB by retrieving and adapting in-vivo timings reported in [20] and [18] studies. Displacement amplitudes of the actuators were gradually set until a qualitatively satisfactory configuration was achieved (by comparison with normal swallowing VFS recordings).

### Evaluation of experiments

In the present study, our objective was to visually demonstrate that Swall-E robot is capable of functionally mimicking a normal (healthy) pharyngeal phase of swallowing in less than 1 s. By healthy swallowing we mean that no injected bolus material penetrates into the ‘wrong’ pipe i.e. the laryngotracheal duct (the ‘right’ pipe being the esophageal duct). For this visual demonstration, the rear and lateral high-speed cameras were utilized to record the videos of the conducted experiments and the obtained videos were evaluated thereafter. The evaluation of the videos was carried out by utilizing two qualitative clinical indicators which assess the safety level of a swallowing process in dysphagic patients, detailed below.

- Visual observation of the absence or presence of aspiration in the trachea.
- Pharyngeal Residue Scale (PRS) by Omari et al. [21] which is a clinical qualitative scale to evaluate the post-swallowing residue level as follows: no residues = 1; valleculae only = 2; posterior pharyngeal wall or piriform sinus only = 3; valleculae and posterior pharyngeal wall or piriform sinus = 4; posterior pharyngeal wall and piriform sinus = 5; and all structures = 6. Note: anatomical locations of the valleculae and piriform sinus are illustrated on Fig 6.

For a normal swallowing sequence, we expect to observe a transit of injected bolus from the oropharyngeal cavity down to the esophagus without any tracheal aspiration, and presence of post-swallowing pharyngeal residues at worst only in the valleculae (i.e. PRS = 2) as observed by Omari et al. in healthy subjects [21].

### Experimental results

**Fig 9:**
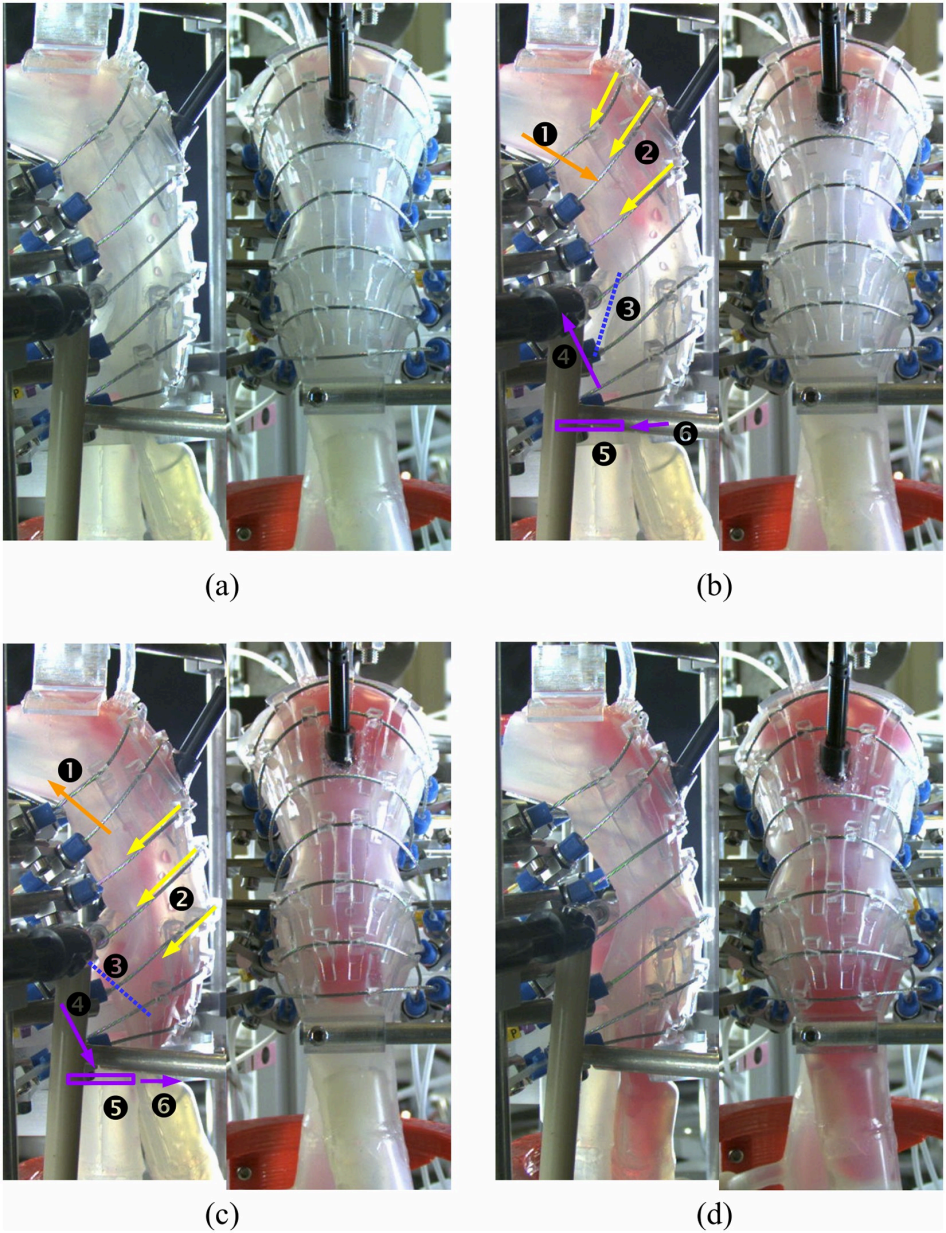
Video captures recorded by lateral and rear high-speed cameras of a normal pharyngeal swallowing sequence of 10 ml tick bolus reproduced by Swall-E robot. (a) [0 ms] Bolus injection in the oropharyngeal cavity. (b) [250 ms] Tongue base push (1); upper pharyngeal contraction (2); initiation of epiglottic tilt (3); initiation of laryngeal elevation (4); adduction (closure) of vocal folds (5) and UES opening (6). (c) [500 ms] Initiation of tongue base retraction (1); middle pharyngeal contraction (2); epiglottis fully tilted (3); initiation of laryngeal descent (4); vocal folds adducted/closed (5) and UES closing (6). (d) [1000 ms] Bolus entering the esophagus; resetting of actuators.

Fig 9 provides some successive video recording captures acquired by the lateral and rear high-speed cameras with corresponding description of occurring mechanisms. Corresponding video files are provided as supporting information (S1 and S2 Videos). We were able to achieve a pharyngeal transit of injected bolus from the injection nozzle outlet located upstream from the oropharyngeal cavity down to the upper esophagus in less than 1 s. No tracheal aspiration of the injected bolus was observed during the whole sequence and after, for at least 2 minutes. However, some residues of bolus material could be observed after the passage of bolus, on the pharyngeal wall, in the valleculae and seemingly in the piriform sinus; i.e. a PRS score of 6. These traces are probably due to too weak bolus propulsion forces [22] and/or the high stickiness of the prepared bolus material as well as a certain lack of lubrication of the anatomical silicone conduit, compared to real pharyngeal walls which are normally lubricated by saliva and mucus. Despite a likely insufficient bolus propulsion, a safe swallowing process without any tracheal aspiration was consistently achieved. Other types of bolus materials and textures will be investigated in future experiments.

### Discussion

The performed normal swallowing sequences demonstrate the ability of our system to translate the chronological order of biomechanical movements from clinical observations into a model where the movement of the food bolus can be physically mimicked and monitored. The availability of such a tool would be beneficial in several aspects. i) The contribution to potential reduction of animal experiments, as there are no relevant animal models for monitoring of swallowing. ii) Obtaining clinically relevant data without patient participation: and the current methods of clinical tests are discomforting for patients with swallowing disorders. iii) In-depth studies of specific swallowing disorders without extensive testing with patients: the available chronograms can be fine-tuned and can be run many times for elucidating the mechanisms of specific swallowing disorders. One main limitation of the system is limited information on the food bolus interaction with the internal surface of the tissue model, which we aim to overcome in the future by incorporating specific sensors. The fouling of the internal surface due to the food remnants, which can have effects on subsequent tests, will be tackled by application of coatings that will enable the elimination of the food residues without having an effect on swallowing sequences.

## Conclusion

### Summary

In this study, we presented a robotic device that can mimic the pharyngeal phase of physiological human swallowing. The potential outlook for such a device is a mean to generate big data related to normal and pathological swallowing, that can bridge gaps in the current level of fundamental knowledge, as the only means of obtaining such information are generally invasive medical procedures. Swall-E has the potential to provide new insights for clinicians, food industry and speech language therapists.

### Perspectives

Our goals with the future iterations of Swall-E are: i) The development and validation of disease specific chronograms and demonstration of aspiration mechanisms similar to those observed clinically for testing potential therapeutic solutions (such as implants) or studying disease mechanisms (such as more precise sub-classification of swallowing disorders not just based on causing diseases (such as stroke) but also with respect to the final aspiration mechanism. ii) Testing of modified food used by dysphagia patients and determination of the most suitable modified food for a given conditions based on swallowing tests in Swall-E. iii) A supporting tool for the rheological studies of food and particularly modified food in a physiologically relevant model.

## Supporting information

S1 Video Lateral view. Swallowing experiment on Swall-E filmed by lateral high-speed camera.
(MP4)

S2 Video Rear view. Swallowing experiment on Swall-E filmed by rear high-speed camera.
(MP4)
